# THE USE OF ARTIFICIAL INTELLIGENCE TRANSCRIPTION TECHNOLOGY IN CAREGIVER QUALITATIVE DATA COLLECTION

**DOI:** 10.1093/geroni/igae098.1492

**Published:** 2024-12-31

**Authors:** Debra Dobbs, Jessica Yauk, Hongdao Meng, Tanjina Jalil

**Affiliations:** University of South Florida, Tampa, Florida, United States; University of South Florida, Tampa, Florida, United States; University of South Florida, Tampa, Florida, United States; University of South Florida, Tampa, Florida, United States

## Abstract

In recent years the use of artificial intelligence (AI) transcription has become more common and has advanced the ability of data collection methods in aging research. This paper presentation includes examples of how AI transcription methods can be implemented into research in multiple ways that has advanced the time to analysis of data and interpretation of qualitative data. The first study example is AI transcription using talk-to-text with an iphone to transcribe handwritten homework assignments from direct caregivers in assisted living in a palliative care education intervention. The second study example is using voice memo AI audio function for assisted living staff interviews in a music group intervention study. The third study uses Microsoft Teams to interview caregivers about advance care planning and end-of-life care quality which produces using AI transcription a transcript of the recorded interview. For each of these AI transcription techniques used in the three studies, we will discuss the advantages and disadvantages of their utilization in qualitative data collection and analysis including the amount of time and money saved in budgeting for transcription costs and whether it is balanced out by the costs for the technology; issues of privacy and older participants’ willingness to allow researchers to use technologies such as smart phones and web-based platforms (e.g., Microsoft Teams and Zoom). We will also discuss the role of technological advancements in qualitative data methods as it relates to the need for technology skill development, privacy and data security.

